# Novel Locus for Paroxysmal Kinesigenic Dyskinesia Mapped to Chromosome 3q28-29

**DOI:** 10.1038/srep25790

**Published:** 2016-05-13

**Authors:** Ding Liu, Yumiao Zhang, Yu Wang, Chanjuan Chen, Xin Li, Jinxia Zhou, Zhi Song, Bo Xiao, Kevin Rasco, Feng Zhang, Shu Wen, Guoliang Li

**Affiliations:** 1 Department of Neurology, the Third Xiangya Hospital, Central South University, Changsha, Hunan, China; 2 Department of Rehabilitation, the Second Hospital of Hebei Medical University, Hebei, China; 3 Comprehensive Epilepsy Center, Department of Neurology, University of Michigan, Ann Arbor, Michigan, USA; 4 Department of Neurology, The First Hospital of Changsha, Changsha, Hunan, China; 5 Key Laboratory of Genome Sciences and Information, Beijing Institute of Genomics, Chinese Academy of Sciences, Beijing, China; 6 Department of Neurology, Xiangya Hospital, Central South University, Changsha, Hunan, China; 7 Department of Molecular and Human Genetics, Baylor College of Medicine, Houston, Texas, USA

## Abstract

Paroxysmal kinesigenic dyskinesia (PKD) is characterized by recurrent and brief attacks of dystonia or chorea precipitated by sudden movements. It can be sporadic or familial. Proline-Rich Transmembrane Protein 2 (*PRRT2*) has been shown to be a common causative gene of PKD. However, less than 50% of patients with primary PKD harbor mutations in *PRRT2*. The aim of this study is to use eight families with PKD to identify the pathogenic *PRRT2* mutations, or possible novel genetic cause of PKD phenotypes. After extensive clinical investigation, direct sequencing and mutation analysis of *PRRT2* were performed on patients from eight PKD families. A genome-wide STR and SNP based linkage analysis was performed in one large family that is negative for pathogenic *PRRT2* mutations. Using additional polymorphic markers, we identified a novel gene locus on chromosome 3q in this *PRRT2*-mutation-negative PKD family. The LOD score for the region between markers D3S1314 and D3S1256 is 3.02 and we proposed to designate this locus as Episodic Kinesigenic Dyskinesia (EKD3). Further studies are needed to identify the causative gene within this locus.

Paroxysmal kinesigenic dyskinesia (PKD) is characterized by attacks of dystonia, chorea or athetosis lasting seconds to minutes. It can either be sporadic or familial. The familial form is often autosomal-dominant with incomplete penetrance^1^.

PKD has been described for over 70 years, which occurs around the world and across many ethnic groups^3^. The pathophysiology of PKD remains unclear. A potential relationship of PKD to epilepsy has been suggested, given the paroxysmal character, short duration of attacks, and co-occurrence of clinical diagnosis. Favorable response to antiepileptic drugs has also been observed in PKD patients^2^. Two partially overlapping PKD loci on chromosome 16, designated as Episodic Kinesigenic Dyskinesia 1 (EKD1^4^; MIM #128200) and Episodic Kinesigenic Dyskinesia 2 (EKD2^5^; MIM #611031), have been previously reported in familial PKD^6^,^7^,^8^,^9^. Recently, Proline-Rich Transmembrane Protein 2 (*PRRT2*) was considered as a PKD causative gene due to its overlapping location to EKD1/EKD2 region^5^. However, less than 50% of patients with primary PKD^10^ have *PRRT2* mutations. Here, we sequenced *PRRT2* in a cohort of eight independent Chinese PKD families and within one large PKD family negative for *PRRT2* mutations (which was referred to as “Family A” below), we identified a novel PKD locus by linkage analysis, and we proposed to designate this novel PKD locus as Episodic Kinesigenic Dyskinesia 3 (EKD3).

## Methods

This study has been approved by institutional review board committee at Xiangya Hospital. Informed written consent was acquired from each participant and family members. All experimental procedures were carried out based on the guidelines approved by the Medical Ethics Committee of Xiangya Hospital.

Clinical evaluation and diagnosis of PKD were conducted based on the guideline proposed by Bruno *et al.*^11^. All affected individuals and their available healthy first-degree relatives were independently evaluated by at least two neurologists in the Department of Neurology, Xiangya Hospital, Central South University, China. Electronic medical records, including demographics, medical history, physical exam, diagnostic procedures and treatment were thoroughly reviewed. The majority of patients had electroencephalogram (EEG), brain magnetic resonance imaging (MRI) and head computed tomography (CT).

The primers, PCR reactions, and *PRRT2* sequencing have been previously described^12^. Controls included were 100 healthy Chinese to determine the frequencies of detected *PRRT2* variations in ethnicity-matched general population.

### Genotyping for linkage analysis

“Family A”, the largest *PRRT2*-mutation negative family in our cohort, was studied by genome-wide linkage analysis. Genotyping was performed in Shanghai at the Chinese National Human Genome Center. Genomic DNA was isolated from whole blood cells with the use of a QIAamp DNA Blood Kit (QIAGEN GmbH, Hilden, Germany). We performed genome-wide scanning using short tandem repeats (STRs) marker with ABI Prism Linkage Mapping Set-MD10 (Applied Biosystems, USA). This kit used 402 polymorphic microsatellite markers with an average spacing of 10 centimorgan (cM). An alternative linkage analysis using single-nucleotide polymorphisms (SNPs) marker was performed with Linkage-12 DNA Analysis Kit (Illumina, USA)^13^. 6,090 SNPs were used with an average spacing of 0.58 cM. Parametric and nonparametric linkage were analyzed with Merlin (version 1.01)^14^. Candidate regions identified by two linkage analyses were then compared and combined.

To further define the candidate region identified by linkage analyses, we performed genotyping and haplotype analyses in “Family A” using 9 STRs (3 were from the original ABI STR kit; and 6 additional ones were added manually from the Marshfield and deCODE genetic maps) within this region. These STRs included D3S3686, D3S1580, D3S1314, D3S1601, D3S3669, D3S2305, D3S240, D3S1265, and D3S1311. Their corresponding physical positions are in supplement Table 1.

## Results

### Identification of *PRRT2* mutations in 8 PKD families

**Figure 1:**
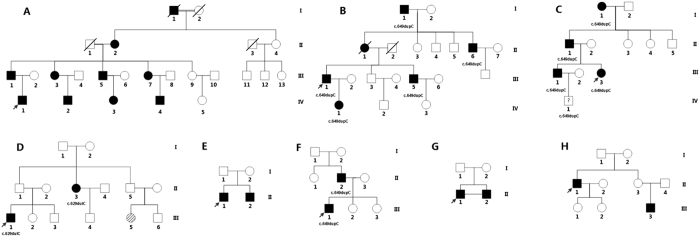
Pedigrees of PKD families and *PRRT2* mutations. Males were represented by squares, and females by circles. Filled-in symbols indicated PKD symptomatic, empty circles indicated unaffected individuals. Symbols with a slash through them indicate deceased individuals. Probands are denoted by an arrow.

After sequencing *PRRT2* in 30 PKD patients in this cohort, we detected two different heterozygous mutations in four of the eight families (Fig. 1, Table 1). The most common *PRRT2* mutation, NM_001256442.1(PRRT2_v001):c.649dup [NM_001256442(PRRT2_i001):p.(Arg217Profs*8)], was identified in families B, C and F of the eight PKD families. The second mutation, NM_001256442.1(PRRT2_v001):c.629dup [NM_001256442(PRRT2_i001):p.(Ala211Serfs*14)], was identified in family D. Both mutations have been previously reported in other ethnic groups and were predicted to result in mRNA degradation by non-sense mediated decay^15^,^16^,^17^,^18^,^19^. None of these two mutations were detected in our 100 control individuals or in the1000 Genome database. The clinical and mutation information were summarized in Table 1. However, no *PRRT2* mutations were identified in family A, E, G, and H. Family A (consisting of 10 patients) is the largest family in our cohort and further investigation were then carried out.

### Description of “Family A”

“Family A” was the largest Chinese PKD family in our cohort. All ten patients in this four-generation pedigree had typical PKD with the autosomal-dominant inheritance pattern (Fig. 1). Attacks of all affected family members were provoked by sudden actions, such as standing up quickly from a sitting position or moving suddenly. Other triggers included nervousness and fatigue. The nature of their attacks can be described as pure dystonia lasting 30 to 60 seconds. No other neurological disorders, such as epilepsy, were identified except for subject IV-5. This male had generalized tonic-clonic seizures following a head trauma at age 12 (Table 1).

**Figure 2:**
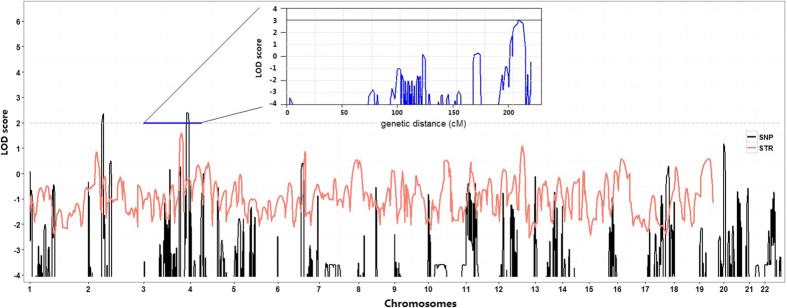
Genome-wide linkage analysis of Family A with PKD. The black and red lines denote LOD values based on SNPs or STRs, respectively. Fine map of Chromosome 3 loci with the addition of six extra STR markers is inset in blue.

Genome-wide STRs scanning showed no significant linkage at EKD1 and EKD2 (LOD score < −3) loci in this family (Fig. 2). Instead, two-point analysis generated a maximum LOD score of 1.75 for marker D3S1580 at recombination fraction of 0. Multipoint linkage analysis also showed a maximum LOD score of 1.60.

Genome-wide SNPs array linkage analysis within this family yielded similar results with higher LOD score. Four continuous SNPs, rs538338, rs3864005, rs1559018 and rs2048417, uniformly showed the highest genome-wide LOD score of 2.399 (Fig. 2). Their corresponding physical positions are in supplement Table 1. They marked a region 204.12 cM–206.38 cM in chromosome 3q28-29. This 2 cM area was 1 cM downstream of D3S1580, the marker with highest score from STRs linkage analysis.

In the next step, by use of manually selected 6 additional markers (D3S3686, D3S1580, D3S1314, D3S1601, D3S3669, D3S2305, D3S240, D3S1265, and D3S1311), linkage locus was narrowed. The maximum interval between these markers was 4.88 cM, with an average of 2.63 cM. Our analysis generated a maximum LOD score of 3.02 in the vicinity of D3S3669 using the same model (Fig. 2). The LOD value exceeded genome-wide significance, indicating a less than 1 to 1000 probability that this linkage is observed by chance.

**Figure 3:**
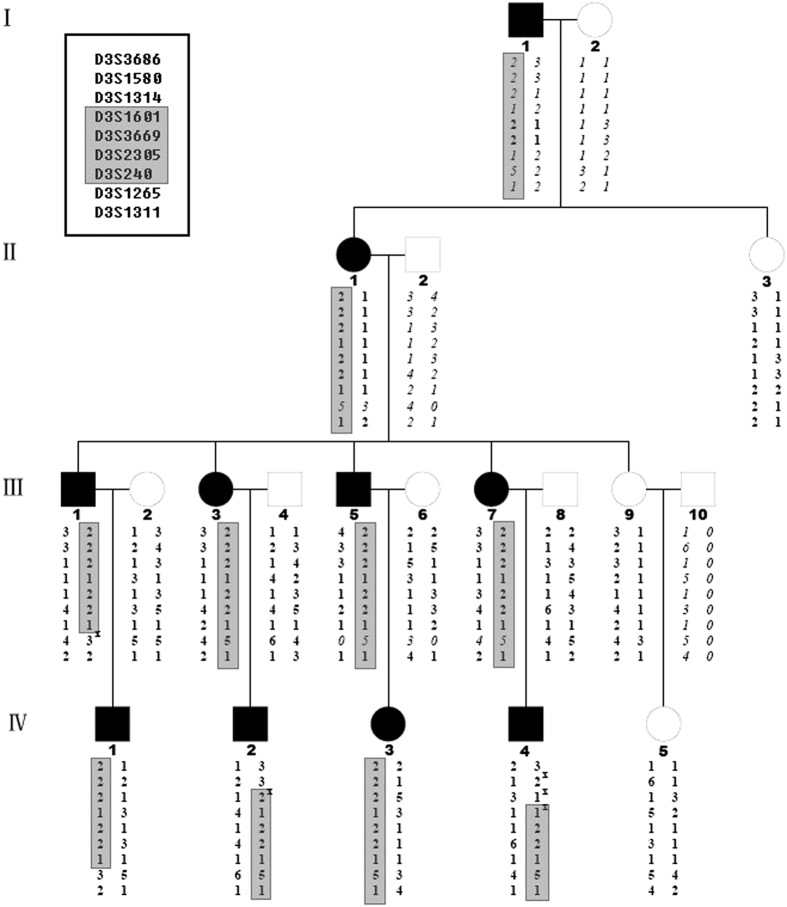
Haplotype Analysis of Family A without *PRRT2* gene mutation. The blackened squares and circles denote individuals affected with PKD. Chromosome 3 loci are indicated. Reconstructed haplotypes using these loci are shown under each individual. Grey bars corresponded to the proposed affected haplotype. X indicates a meiotic recombination event.

Haplotypes were constructed to determine the critical recombination events. Recombination events in IV-1 and IV-4 allowed the candidate region of PKD locus to be narrowed down to a 5.6 cM interval between markers D3S1314 and D3S1265. A common haplotype, 1-2-2-1 for markers D3S1601, D3S3669, D3S2305 and D3S240, is shared by all ten affected members but was absent in any of the eight healthy family members (Fig. 3). The data provided strong evidence for PKD susceptibility loci outside of the previously defined regions EKD1/2 on chromosome 16.

## Discussion

We have identified *PRRT2* mutations in four of the eight PKD families (50%), similar to the previous reports^10^. The two mutations identified in our study have both been reported before^20^. These findings suggest that Chinese PKD patients share common genetic characteristics with patients from other ethnic groups, which can be explained by the ancient founder-effect, or these loci might be mutation hotspots. Family A, negative for *PRRT2* mutations, is the largest PKD pedigree that has been reported to date. By using the STR kit with an average of 10 cM marker interval, we found the highest LOD score of 1.75 at 3q28-29.SNPs markers, although with lower heterogeneity, have much higher density compared to STRs markers. Thus, when some parental information was missing, as was the case in Family A, including SNPs markers could increase statistical power by up to 20%^21^. SNP array linkage analysis did confirm the linkage in this region with a higher LOD score of 2.399 for four contiguous SNPs markers. Additionally, we manually added six STR markers into this region. Subsequent haplotype analysis showed a 5.6 cM linkage region between the markers D3S1314 and D3S1265, with a LOD score of 3.02, exceeded genome-wide significance. This region is shared by all affected members but is absent in all healthy members in this family (see Fig. 1). Therefore, we propose this novel linkage locus as EKD3, following the current nomenclature assignment for PKD loci.

Interestingly, this region has previously been linked to familial epilepsy, a disease that may have common pathophysiology with PKD^22^,^23^. Hirata *et al.*^24^ reported generalized rhythmic 5Hz discharges during episodes of PKD. Beaumanoir *et al.*^25^ documented a PKD patient after a prolonged attack, which induced a postictal state and impaired consciousness. In addition, infantile convulsions and paroxysmal dyskinesia (ICCA) manifests as infantile epilepsy followed by childhood paroxysmal dyskinesias^26^. *PRRT2* has been recently identified as the cause of three clinical entities: benign familial infantile epilepsy (BFIE), infantile convulsions with choreoathetosis (ICCA) syndrome, and PKD^27^. Although there may be an overlap between these two syndromes with unknown causes of pathophysiology or genetics, these reasons are not sufficient to consider PKD as simply another form of epilepsy. Our findings therefore support a possible common genetic etiology in PKD and other forms of epilepsy^28^. Our data also suggest that susceptibility variants for both syndromes may be found within the EKD3 locus.

Several candidate genes are located in this region. Given the gradual onset in childhood or adolescence, as well as the gradual remission after the third decade of age, genes that influence brain development should be considered, such as Cation-Transporting ATPase 13A3 (*ATP13A3*)^29^ and Protein Kinase C Epsilon Type (*PRKCE*), which have been reported to regulate calcium channels^30^. Other genes encoding ion channels in brain, including Potassium Voltage-Gated Channel Subfamily G member 3 (*KCNG3*) and Solute carrier family 8 [sodium/calcium exchanger] Member 1(*SLC8A1*), are also possible candidate genes for PKD.

In summary, we report the clinical and genetic characteristics of eight Chinese PKD families, and have provided strong evidence for the linkage of familial PKD to chromosome 3q28-29 within a large Chinese PKD family. This locus is distinct from any previously reported PKD regions, and thus we propose the designation, “EKD3”. The present study further supports the hypothesis that more than one PKD causative gene exist. Further investigation will be necessary to confirm the significance of EKD3 with more PKD pedigrees in order to elucidate the causative genes, pathways, and molecular mechanisms by which genetic mutations lead to these alterations in human neural circuitry.

## Additional Information

**How to cite this article**: Liu, D. *et al.* Novel Locus for Paroxysmal Kinesigenic Dyskinesia Mapped to Chromosome 3q28-29. *Sci. Rep.* **6**, 25790; doi: 10.1038/srep25790 (2016).

## Supplementary Material

Supplementary Information

## Figures and Tables

**Table 1:** Clinical Features and *PRRT2* mutations in 30 affected members from 8 Chinese PKD families.

Family	Subject	Age(y)	Gender	Age at Onset(y)	Attack frequency	Attack duration	Triggers	Involuntary movement	Previous history	Response to anticonvulsants	EEG	CT/MRI	*PRRT2* mutations
A	I-1	–	M	15	1	<10s	SM	D	no	No TMT	NA	NA	–
	II-1	88	F	14	1–2/y, 3 y	<5s	SM	D	no	No TMT	–	–	–
	III-4	60	F	12	2–3/d	<10s	SM	D	no	No TMT	–	–	–
	III-6	58	F	9	2–4/d	30–60s	SM,S	D	no	No TMT	–	–	–
	III-7	56	M	11	3–4/d	10–30s	SM	D	no	No TMT	–	–	–
	III-9	54	M	12	5–10/d	<10s	SM	D	no	No TMT	–	–	–
	IV-2	35	M	8	5–10/d	30–60s	SM	D	no	No TMT	–	–	–
	IV-3	31	M	8	>30/d	30–60s	SM	D	no	No TMT	–	–	–
	IV-4	26	F	8/19	10–20/d	10–30s	SM	D	no	CBZ(+)	–	–	–
	IV-5	26	M	4	20–30/d	<10s	SM,S	D	EP	VPA(+)	+	–	–
B	I-1	88	F	30	3–4/d	<10s	SM	D/C	no	No TMT	–	–	c.649dup
	II-1	–	F	18	3–5/d	<10s	SM	C	no	No TMT	–	–	NA
	II-6	43	M	15	5–10/d	<10s	SM	C	no	No TMT	–	–	c.649dup
	III-1	43	M	13	5–10/d	<10s	SM	C	no	No TMT	–	–	c.649dup
	III-5	39	M	10	>30/d	<10s	SM	C	no	CBZ(+)	–	–	c.649dup
	IV-1	22	F	7	10–20/d	<30s	SM	C	no	CBZ(+)	–	–	c.649dup
C	I-1	76	F	10	0–1/m	5–10s	SM	D/C	no	No TMT	NA	NA	c.649dup
	II-1	50	M	7	0–5/d	8–10s	SM	D/C	no	No TMT	NA	NA	c.649dup
	III-1	28	M	8	3–4/d	5–10s	SM	C	no	No TMT	NA	NA	c.649dup
	III-3	24	F	6	1–5/d	10–20s	SM, Ex	D/C	no	CBZ(+)	–	–	c.649dup
D	II-3	42	F	15	0–2/d	5–10s	SM	D/C	no	No TMT	–	–	c.629dup
	III-1	26	M	13	1–3/m	5–20s	SM,S	C	IC	CBZ(+)	–	–	c.629dup
E	II-1	28	M	13	0–5/d	3–5s	SM	D	no	CBZ(+)	–	–	–
	II-2	24	M	11	10–20/d	3–5s	SM	D	no	CBZ(+)	–	–	–
F	II-2	57	M	15	1–2/y	0–10s	SM	D/C	no	No TMT	–	–	c.649dup
	III-1	29	M	7–8	20–30/d	0–10s	SM	D/C	no	CBZ(+)	–	–	c.649dup
G	II-1	15	M	10	3–10/d	10–20s	SM	D	no	CBZ(+)	–	–	–
	II-2	15	M	10	3–10/d	10–20s	SM	D	no	CBZ(+)	–	–	–
H	II-1	41	M	12	3–5/d	0–10s	SM	D	no	No TMT	–	–	–
	III-3	16	M	11	10–20/d	0–10s	SM	D	no	CBZ(+)	–	–	–

M: male; F: female; d: day; m: month; y: year; s: seconds; SM: sudden movement; Ex: exertion; S: surprise; D: dystonia; C: chorea; EEG: electroencephalogram; CT: computed tomography; MRI: magnetic resonance imaging; IC: infantile convulsions; EP: epilepsy; NA: not available; +: positive; −: negative; CBZ: carbamazepine; VPA: valproate; No TMT: No Treatment.
