# Supplementary material for: Novel Locus for Paroxysmal Kinesigenic Dyskinesia Mapped to Chromosome 3q28-29
Source: Sci Rep. 2016 May 13;6:25790. doi: 10.1038/srep25790 (PMC4865737; doi:10.1038/srep25790)
Supplement: Supplementary Information [file srep25790-s1.pdf]

# Novel Locus for Paroxysmal Kinesigenic Dyskinesia Mapped to Chromosome 3q28-29

Ding Liu[1,*], Yumiao Zhang[3, *]; Yu Wang[4], Chanjuan Chen[5], Xin Li[7], Jinxia Zhou[2], Zhi Song[1], Bo Xiao[2], Kevin Rasco[6], Feng Zhang[7], Shu Wen[6], Guoliang Li[2]

[1]Department of Neurology, the Third Xiangya Hospital, Central South University, Changsha, Hunan, China.

[2]Department of Neurology, Xiangya Hospital, Central South University, Changsha, Hunan, China.

[3]Department of Rehabilitation, the Second Hospital of Hebei Medical University.

[4]Comprehensive Epilepsy Center, Department of Neurology, University of Michigan, Ann Arbor, Michigan, USA

[5]Department of Neurology, The First Hospital of Changsha, Changsha, Hunan, China

[6]Department of Molecular and Human Genetics, Baylor College of Medicine, Houston, Texas, USA.

[7]Key Laboratory of Genome Sciences and Information, Beijing Institute of Genomics, Chinese Academy of Sciences, Beijing, China

[*]These authors contributed equally to this work.

Physical positions of relevant SNPs and Markers in the linked region

| | |
|---|---|
| rs538338 | chr3:190166614 |
| rs3864005 | chr3:190304764 |
| rs1559018 | chr3:190579044 |
| rs2048417 | chr3:191064484 |
| D3S3686 | chr3:187699669-187700079 |
| D3S1580 | Chr3:188825005-188825348 |
| D3S1314 | chr3:190374086-190374451 |
| D3S1601 | chr3:191959487-191959819 |
| D3S3669 | chr3:192784176-192784525 |
| D3S2305 | chr3:193712492-193712994 |
| D3S240 | chr3:194362025-194362239 |
| D3S1265 | chr3:195799159-195799433 |
| D3S1311 | chr3:197291105-197291428 |
